# Rapid Decline of Serum Proprotein Convertase Subtilisin/Kexin 9 (PCSK9) in Non-Cirrhotic Patients with Chronic Hepatitis C Infection Receiving Direct-Acting Antiviral Therapy

**DOI:** 10.3390/jcm10081621

**Published:** 2021-04-11

**Authors:** Jonathan Grimm, Georg Peschel, Martina Müller, Doris Schacherer, Reiner Wiest, Kilian Weigand, Christa Buechler

**Affiliations:** 1 Department of Internal Medicine I, Gastroenterology, Hepatology, Endocrinology, Rheumatology and Infectious Diseases, University Hospital Regensburg, 93053 Regensburg, Germany; jonathan.grimm@stud.uni-regensburg.de (J.G.); georg.peschel@klinik.uni-regensburg.de (G.P.); martina.mueller-schilling@klinik.uni-regensburg.de (M.M.); doris.schacherer@barmherzige-regensburg.de (D.S.); kilian.weigand@klinik.uni-regensburg.de (K.W.); 2 Department of Visceral Surgery and Medicine, University Inselspital, 3010 Bern, Switzerland; Reiner.Wiest@insel.ch

**Keywords:** low-density lipoprotein, liver cirrhosis, Proprotein convertase subtilisin/kexin 9 (PCSK9), hepatitis C, MELD score, genotype

## Abstract

Direct-acting antivirals (DAAs) efficiently eradicate the hepatitis C virus (HCV). Low-density lipoprotein (LDL) levels increase rapidly upon DAA treatment. Proprotein convertase subtilisin/kexin 9 (PCSK9) induces degradation of the hepatic LDL receptor and thereby elevates serum LDL. The aim of this study was to determine serum PCSK9 concentrations during and after DAA therapy to identify associations with LDL levels. Serum PCSK9 was increased in 82 chronic HCV-infected patients compared to 55 patients not infected with HCV. Serum PCSK9 was low in HCV patients with liver cirrhosis, but patients with HCV-induced liver cirrhosis still exhibited higher serum PCSK9 than patients with non-viral liver cirrhosis. Serum PCSK9 correlated with measures of liver injury and inflammation in cirrhotic HCV patients. In patients without liver cirrhosis, a positive association of serum PCSK9 with viral load existed. Serum PCSK9 was not different between viral genotypes. Serum PCSK9 did not correlate with LDL levels in HCV patients irrespective of cirrhotic status. Serum PCSK9 was reduced, and LDL was increased at four weeks after DAA therapy start in non-cirrhotic HCV patients. Serum PCSK9 and LDL did not change upon DAA treatment in the cirrhotic group. The rapid decline of PCSK9 after the start of DAA therapy in conjunction with raised LDL levels in non-cirrhotic HCV patients shows that these changes are not functionally related.

## 1. Introduction

Chronic hepatitis C virus (HCV) infection is a risk factor for liver fibrosis, cirrhosis and hepatocellular carcinoma [1]. HCV is successfully eliminated by the use of direct-acting antivirals (DAA), and sustained virologic response (SVR) of up to 100% can be achieved [2]. HCV eradication normalizes serum levels of aminotransferases and ferritin, whereas bilirubin and albumin levels are barely changed [3,4,5,6,7]. DAA therapy results in short-term improvements of hepatic inflammation but not fibrosis [8].

Of note, HCV reprograms the host lipoprotein metabolism for the production of viral particles. Indeed, expression of the LDL-receptor was increased in HCV-infected livers [9,10]. Accordingly, serum cholesterol and low-density lipoprotein (LDL) levels were low in patients with chronic HCV infection [11,12]. Upon DAA therapy, serum LDL rapidly recovers [5,13], showing that lipid metabolism’s perturbation is a direct effect of HCV infection.

Proprotein convertase subtilisin/kexin 9 (PCSK9) is an enzyme predominantly produced by the liver [14]. Hepatic LDL-receptor protein is degraded upon PCSK9 binding, and serum LDL is increased. Blockage of PCSK9 lowers serum LDL and may protect from cardiovascular diseases [15,16,17].

HCV uses the LDL-receptor to enter hepatocytes [18,19]. PCSK9 participates in the degradation of the LDL-receptor and probably has a role in HCV infection [18,19]. Indeed, an inhibitory function of PCSK9 on HCV infection has been described [18,20]. Alirocumab, a therapeutic human anti-PCSK9 antibody, was, however, therapeutically ineffective [21].

Of clinical relevance, HCV-infected patients had higher serum PCSK9 levels, whereas LDL did not increase in parallel [22]. Serum PCSK9 negatively correlated with the model for end-stage liver disease (MELD) score, which is the state-of-the-art scoring model of liver disease severity [22,23]. In addition, HCV genotype-dependent regulation of plasma PCSK9 levels has been described. PCSK9 concentrations were lower in HCV genotype 3 patients compared to HCV-negative controls. HCV genotype 1-infected patients had higher PCSK9 levels than non-infected controls [24]. PCSK9 did not correlate with LDL in HCV genotype 1- or 3-infected patients [24].

LDL levels rapidly increase during DAA treatment [5,13,25]. Whether PCSK9 is induced in parallel is still poorly understood.

The studies cited above measured total PCSK9 protein by ELISA. PCSK9 is inactivated by proteolysis, and up to 40% of circulating PCSK9 is inactive [26]. One study reported that active PCSK9 declined, and the inactive form increased at week four after the start of daclatasvir/asunaprevir therapy in patients infected with HCV genotype 1b. At 24 weeks after the start of therapy, active and inactive PCSK9 concentrations returned to pretreatment levels. At 28 weeks posttreatment, active PCSK9 was higher compared to levels before therapy starts. Inactive PCSK9 was not changed during this period. In the serum of HCV patients, levels of inactive PCSK9 were about five-fold lower than the concentration of the active isoform [27]. Thus, total PCSK9 declined at four weeks after therapy start and steadily increased afterward. LDL was induced from week four after the start of therapy to six months posttreatment. These authors suggested that active PCSK9 has a role in the recovery of LDL. Active PCSK9 and LDL levels did not correlate before therapy and at 52 weeks posttreatment [27].

The association of serum PCSK9 with HCV infection and HCV-related dyslipidemia is not completely understood. Here, serum PCSK9 was measured in controls and patients with chronic HCV infection and the effect of HCV eradication on serum PCSK9 was determined.

## 2. Materials and Methods

### 2.1. Study Cohort

This study was conducted at the University Hospital of Regensburg from October 2014 to September 2019. Patients older than 18 years with chronic hepatitis C participated in the study. Treatment naive patients eligible for DAA therapy based on the EASL guidelines were included [2]. Patients were excluded if they were coinfected with HBV or HIV.

Statin taking was stopped during therapy. Most of the patients were treated with DAAs early after approval of these therapies, and at that time, the drug–drug interaction of statins and DAAs was suggested to be higher than known today [28].

Cirrhosis diagnosis by ultrasound was based on coarse liver parenchyma, nodular liver surface, and small liver size [29].

The fasting serum of the patients was collected in the morning. Serum aliquots were stored at −80 °C. Routine laboratory parameters were determined at the Institute of Clinical Chemistry and Laboratory Medicine (University Hospital Regensburg). All patients had chronic hepatitis C and were treated with one of the following regimens: sofosbuvir/daclatasvir, sofosbuvir/ledipasvir, glecaprevir/pibrentasvir, elbasvir/grazoprevir or sofosbuvir/velpatasvir, following international treatment guidelines [2].

The controls included patients referred to ultrasound imaging. Patients with cancers, severe liver diseases or liver cirrhosis were excluded. Data on PCSK9 serum levels in this control cohort were published before and are retrieved in this analysis for comparison with HCV-infected patients [10]. Moreover, analysis of PCSK9 in patients with predominant alcoholic liver cirrhosis was performed before, and data of patients with the non-viral disease were reused in this manuscript [10].

Patients’ characteristics are given in Table 1 and Table 2. All patients gave informed consent prior to inclusion in the study.

### 2.2. PCSK9 ELISA

The human PCSK9 DuoSet ELISA was obtained from R&D Systems (Wiesbaden, Nordenstadt, Germany) and was performed as requested by the provider (dilution of serum was 1:100). The serum used for analysis was thawed twice. The inter-assay coefficient of variation, which was determined from three independent experiments, was 3.4% for high values (serum of 8 patients diluted 1:10), 3.5% for median values (serum of 8 patients diluted 1:50) and 6.1% for low values (serum of 8 patients diluted 1:200). The intra-assay coefficient of variation was 4.3% for high values, 4.3% for median values and 7.6% for low values (Supplementary Materials). When serum of three donors was spiked with 1280 pg/mL recombinant PCSK9, recovery was 83%.

### 2.3. Statistical Analysis

Data are shown as boxplots. Small circles or asterisks above or below the boxes mark outliers. Statistical tests used were one-way ANOVA with post hoc Bonferroni, Mann–Whitney *U*-test, Spearman correlation, partial correlation (SPSS Statistics 25.0 program, IBM, Armonk, NY, USA) and *t*-test (Microsoft Excel). Two variables were compared by the chi-squared test. A value of *p* < 0.05 was regarded as significant.

## 3. Results

### 3.1. Serum PCSK9 Is Increased in HCV Patients

Serum PCSK9 was determined in 82 patients with chronic HCV infection. These samples were used recently for analysis of chemerin [5].

**Figure 1:**
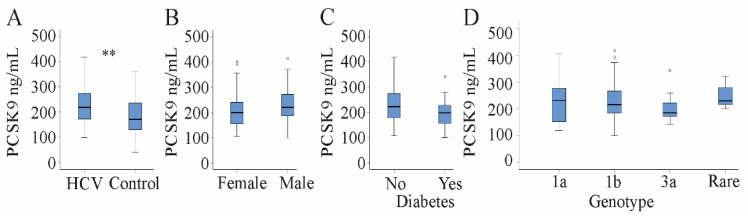
Serum proprotein convertase subtilisin/kexin 9 (PCSK9) in patients with chronic hepatitis C virus (HCV) and controls. (**A**) Serum PCSK9 in patients with chronic HCV and non-infected controls without severe liver diseases; (**B**) serum PCSK9 in female and male HCV patients; (**C**) serum PCSK9 in diabetic and non-diabetic HCV patients; (**D**) serum PCSK9 in patients stratified for HCV genotype (rare group is 6 patients with genotypes other than 1a, 1b, and 3a). Small circles above the boxes mark outliers. ** *p* < 0.01.

Comparison of serum PCSK9 between HCV patients and non-infected patients without any severe liver diseases showed that PCSK9 levels were 170 (40–360) ng/mL in the non-infected controls and 218 (99–418) ng/mL in HCV patients and were significantly higher in the HCV group (Figure 1A). Serum LDL was low in HCV patients compared to the control cohort as was described before [30] (Table 1).

### 3.2. Serum PCSK9 Correlates with Markers of Liver Injury, Viral Load and LDL in HCV Patients

Serum PCSK9 was similar in male and female HCV patients (Figure 1B) and did not correlate with body mass index (BMI) (Table 3). A modest negative association with age existed (Table 3). The 18 diabetic HCV patients had similar PCSK9 levels compared to the non-diabetic patients (Figure 1C).

Serum PCSK9 negatively correlated with bilirubin and INR, and accordingly with the MELD score (which is calculated from bilirubin, INR and creatinine [23]). There were modest negative associations with AST and CRP. Positive correlations existed for leukocyte count and for LDL (Table 3).

Serum PCSK9 was positively associated with viral load before therapy start (Table 3). HCV genotypes were 1a, 1b and 3a in 24, 38 and 14 patients, respectively. Six patients had different genotypes and were assigned to one group. Serum PCSK9 was similar in all of these patients (Figure 1D).

It must be noted that the prevalence of ultrasound diagnosed liver cirrhosis was similar between the different HCV genotypes and was 50% in patients with genotype 1a, 53% in patients with genotype 1b, 36% in patients with genotype 3a and 0% in patients with rare genotypes. 

### 3.3. Serum PCSK9 in Relation to Liver Steatosis, Cirrhosis and Fibrosis Scores

**Figure 2:**
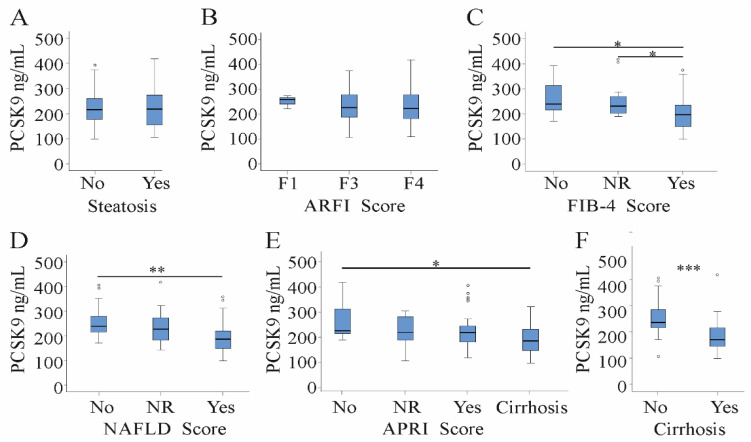
Serum PCSK9 in patients with chronic HCV stratified for steatosis, fibrosis scores and cirrhosis. (**A**) PCSK9 in serum of 37 patients with and 45 patients without liver steatosis; serum PCSK9 levels in patients stratified for fibrosis by (**B**) the acoustic radiation force impulse (ARFI) score; (**C**) the fibrosis-4 (FIB-4) score; (**D**) the non-alcoholic fatty liver disease (NAFLD) score and (**E**) the aminotransferase/platelet ratio index (APRI) score (no fibrosis = no; not reliable values = NR, fibrosis = yes); (**F**) serum PCSK9 in 37 patients with liver cirrhosis and 45 non-cirrhotic patients assessed by ultrasound. Small circles above or below the boxes mark outliers. * *p* < 0.05, ** *p* < 0.01, *** *p* < 0.001.

Thirty-seven HCV patients had liver steatosis as was diagnosed by ultrasound imaging. Serum PCSK9 levels were not changed in patients having the fatty liver disease (Figure 2A). Non-invasive scores assessed liver fibrosis: (1) acoustic radiation force impulse (ARFI) indicated liver fibrosis in 67 patients, (2) the NAFLD score (which integrates age, BMI, diabetes, AST/ALT ratio, platelet count and albumin [31]) in 35 patients, (3) the aspartate aminotransferase/platelet (AST/PLT) ratio index (APRI) in 57 patients, and (4) the fibrosis 4 (FIB-4) score (calculated from age, AST, ALT and platelet count [32,33] in 53 patients (Table 1).

Increasing ARFI fibrosis score did not affect serum PCSK9 levels (Figure 2B). PCSK9 levels negatively correlated with fibrosis assessed by the FIB-4 score, the NAFLD score and the APRI score (Figure 2C–E). Accordingly, serum PCSK9 was 171 (99–418) ng/mL in the 37 HCV patients with liver cirrhosis and 235 (106–406) ng/mL in HCV patients without liver cirrhosis and was strongly reduced in patients with liver cirrhosis diagnosed by ultrasound (Figure 2F).

**Figure 3:**
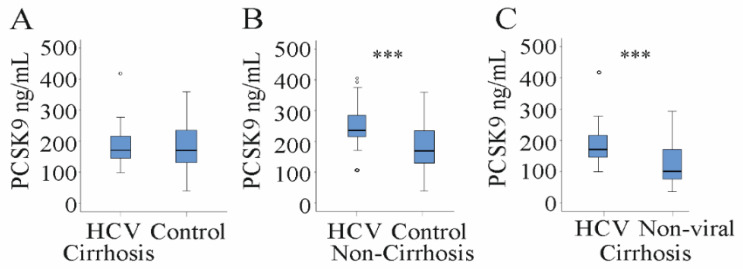
Serum PCSK9 in controls, non-cirrhotic HCV patients and cirrhotic patients with HCV or non-viral disease etiology. (**A**) Serum PCSK9 of HCV patients with liver cirrhosis and controls not infected with HCV; (**B**) Serum PCSK9 of HCV patients without liver cirrhosis and the controls not infected with HCV; (**C**) Serum PCSK9 in patients with HCV and non-viral liver cirrhosis. Small circles above or below the boxes mark outliers. *** *p* < 0.001.

HCV patients with liver cirrhosis indeed had PCSK9 serum levels (171 (99–418) ng/mL) as low as the non-HCV-infected controls (170 (40–360) ng/mL; Figure 3A). Thus, PCSK9 was induced in non-cirrhotic HCV patients with 235 (106–406) ng/mL serum PCSK9 compared to non-HCV-infected controls (170 (40–360) ng/mL; Figure 3B).

Cirrhosis patients with non-viral disease etiology had 108 (41–290) ng/mL PCSK9 in serum (Table 3). Serum PCSK9 was higher in HCV patients with liver cirrhosis (171 (99–418) ng/mL) (Figure 3C, Table 3). This indicates that HCV infection induces serum PCSK9 in cirrhotic and non-cirrhotic patients.

The strong decline of serum PCSK9 in cirrhotic patients (Figure 2F) prompted us to analyze associations of serum PCSK9 and laboratory parameters separately in HCV patients with and without liver cirrhosis.

Of note, negative associations of serum PCSK9 with the MELD score, ALT, bilirubin, INR and CRP and positive correlations with albumin and leukocyte count existed in the HCV patients with liver cirrhosis. Partial correlation corrected for age, BMI, liver steatosis, diabetes and LDL revealed significant associations of serum PCSK9 with the MELD score (*r* = −0.395, *p* = 0.031), bilirubin (*r* = −0.372, *p* = 0.043), leukocyte count (*r* = 0.521, *p* = 0.003) and CRP (*r* = −0.459, *p* = 0.011).

In non-cirrhotic patients, a positive correlation of serum PCSK9 with viral load was noticed (Table 4). This correlation was still significant after correction for age, BMI, liver steatosis, diabetes and LDL (*r* = 0.382, *p* = 0.037). Serum PCSK9 did not correlate with LDL levels in both cohorts (Table 4), suggesting that associations observed in the whole study group were merely based on the differences of serum PCSK9 and LDL between cirrhotic and non-cirrhotic patients (Table 1 and Figure 1A).

### 3.4. DAA Therapy Reduces Serum PCSK9 Levels in Non-Cirrhosis Patients

**Figure 4:**
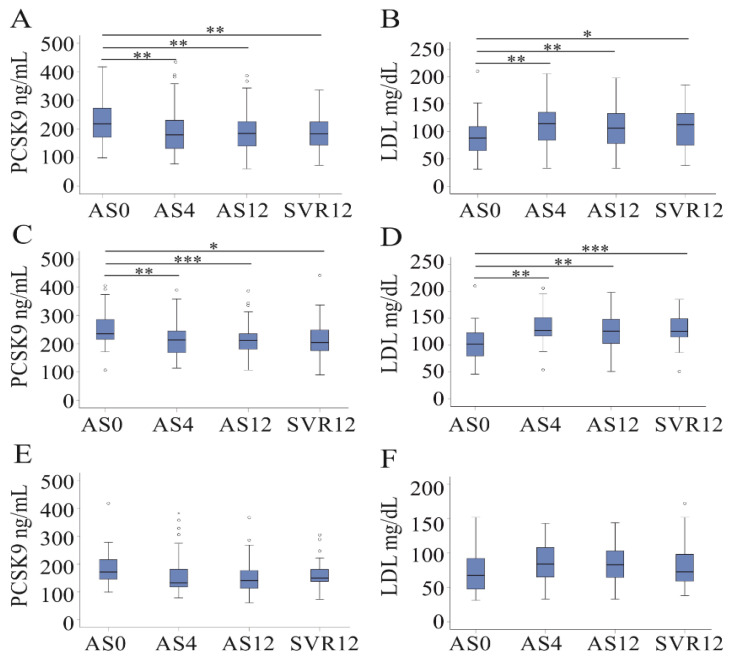
Serum PCSK9 and LDL at 0, 4 and 12 weeks after the start of therapy (AS), and 12 weeks after the end of therapy (SVR12). Serum PCSK9 (**A**) and LDL (**B**) in the whole cohort; serum PCSK9 (**C**) and LDL (**D**) in non-cirrhotic HCV patients; serum PCSK9 (**E**) and LDL (**F**) in cirrhotic HCV patients. Small circles above or below the boxes mark outliers. * *p* < 0.05, ** *p* < 0.01 and *** *p* < 0.001.

PCSK9 was reduced from 218 (99–418) ng/mL to 179 (78–390) ng/mL already at 4 weeks after the start of DAA therapy in the whole cohort (Figure 4A). Recently, we have shown that LDL levels were induced already at 4 weeks after the start of DAA therapy in this cohort [5], and this is illustrated in a diagram (Figure 4B). Because serum PCSK9 was low in liver cirrhotic patients before therapy start (Figure 2F), DAA-treatment-related effects were separately calculated in HCV patients with and without cirrhosis. Serum PCSK9 was 235 (106–406) ng/mL in non-cirrhotic patients before therapy and 213 (114–390) ng/mL at 4 weeks after therapy start (Figure 4C). In the cirrhotic patients, PCSK9 was 171 (99–418) ng/mL before therapy and 132 (78–383) ng/mL at 4 weeks after therapy start (Figure 4E). The decline of PCSK9 during DAA therapy was significant in non-cirrhotic patients (Figure 4C). LDL was induced in non-cirrhotic patients at all of the time points (Figure 4D). In HCV patients with liver cirrhosis, DAA therapy was not associated with higher LDL levels (Figure 4F).

At all of the time points, serum PCSK9 levels were higher in non-cirrhotic than in cirrhotic patients (*p* < 0.001 for 4 and 12 weeks after therapy start and for 12 weeks posttreatment).

At 12 weeks posttreatment, the associations of serum PCSK9 with the MELD score, bilirubin, INR, leukocytes and CRP in the patients with liver cirrhosis persisted. At this time point, a positive correlation of PCSK9 and LDL was observed (Table 4).

A poor correlation of serum PCSK9 with albumin was detected in non-cirrhotic patients at 12 weeks posttreatment (Table 4).

## 4. Discussion

Current analysis showed a weak association of serum PCSK9 with LDL in patients with chronic HCV infection. PCSK9 was low in patients with liver cirrhosis and correlated with measures of liver injury. In non-cirrhotic HCV patients, serum PCSK9 was positively associated with viral load. Serum PCSK9 declined during DAA therapy in non-cirrhotic but not cirrhotic HCV patients. In cirrhotic patients, the impact of liver disease seems to override the effect of viral eradication on serum PCSK9.

Few researchers have compared PCSK9 levels in HCV-infected patients and non-infected controls. Bridge et al. showed that HCV genotype 3 patients had lower and HCV genotype 1 patients had higher plasma PCSK9 levels compared to uninfected controls [24]. It must be noted that plasma and serum PCSK9 levels do not correlate, and it is debatable whether studies using different biofluids can be compared [34]. In HIV-infected patients, coinfection with HCV increased plasma PCSK9 levels [35]. Fasolato et al. observed higher plasma PCSK9 in HCV-infected patients than in non-infected controls [22]. Our study identified elevated PCSK9 levels in serum of patients with chronic HCV compared to non-infected patients without severe liver diseases. This also applies to patients with liver cirrhosis where PCSK9 was higher in HCV patients than patients with non-viral disease etiology. Thus, HCV infection seems to induce serum and plasma PCSK9 and, when comparing different patient cohorts, the severity of liver fibrosis must be considered

PCSK9 declined during prolonged fasting, while levels were not reduced after fasting for 8 to 12 h [36]. Serum PCSK9 levels were moreover induced by dietary saturated fat, fructose and cholesterol [37]. The studies cited above regarding systemic PCSK9 levels in HCV-infected patients used fasting plasma [22,24,35]. Fasting serum was obtained from the patients in the current analysis. A diurnal rhythm, with the lowest PCSK9 levels between 3 and 9 p.m. and a peak at about 4:30 a.m., was also reported [38]. Samples of our cohort were all obtained in the morning, and this may also apply to the studies cited above [22,24,35].

Lipid-lowering drugs were shown to affect circulating PCSK9 levels. Statins increase hepatocyte LDL-receptor and PCSK9 expression, and previous studies described that statins induced systemic PCSK9 concentrations by 6 to 39% [39]. Patients enrolled in the study by Fasolato et al. did not receive statins [22], and statins were temporally discontinued in our patient cohort. The patients analyzed in the study of Kohli et al. and Bridge et al. were not preselected for cardiovascular risk factors, and the use of lipid-lowering drugs was not reported [24,35]. There is some evidence that ezetimibe may also induce PCSK9, whereas fibrates seem to lower its levels [39]. Intake of these drugs is mostly not discontinued during DAA therapy, and the effects of these drugs on circulating PCSK9 may be similar before and after viral eradication. Therefore, it is unlikely that DAA therapy-induced changes of PCSK9 are associated with these medications.

About 18% of the US population was on statin therapy in 2014 [40], and the prevalence of statin use seems to be similar in HCV-infected patients [41]. Thus, higher levels of serum PCSK9 in HCV patients are most likely not a consequence of statin therapy.

HCV genotype appeared to modulate plasma PCSK9 levels in a previous cohort [24] but was not associated with serum PCSK9 in the current analysis. Prevalence of liver cirrhosis and MELD scores did not vary between the genotypes in the present study. The MELD score was not reported for the patients enrolled by Bridge et al. [24] and may be a confounding variable. A second study reported comparable PCSK9 levels in patients with genotype 1a, 1b and 2. Genotype 3 patients had lower PCSK9 levels compared to genotype 2-infected patients. The MELD score was similar between these subgroups [22]. Genotype 2 was rare in the current study cohort, and statistical analysis was, therefore, not possible. However, similar levels of PCSK9 in genotype 1a and 1b-infected patients [22] were following the present observations.

Serum PCSK9 positively correlated with albumin and negatively with the MELD score in our HCV patient cohort, and this is in agreement with a previous analysis [22]. These associations existed in the HCV-infected patients with liver cirrhosis but not in the non-cirrhotic group. Associations of PCSK9 and the MELD score were still significant after correcting for confounding variables. Correlations of PCSK9 and the MELD score could not be observed in non-HCV-infected patients with severe liver diseases [22]. Plasma PCSK9 was not changed with increasing MELD score in liver cirrhotic patients with mostly alcoholic disease etiology [10]. Schlegel et al. nevertheless described a negative correlation of serum PCSK9 with the MELD score in a cohort of patients with severe liver disease and mixed disease etiology. Notably, only 2.7% of these patients were infected with HCV [42]. This suggests that associations of PCSK9 with residual liver function are affected by liver disease severity and disease etiology.

Hepatic PCSK9 protein expression was found increased in the cirrhotic liver, and hepatocellular carcinoma adjacent tissues were used in this analysis. PCSK9 was determined by immunohistochemistry; the fibrosis stage could have been evaluated by histopathology. This study did, however, not discriminate between HCV-infected patients and patients with other disease etiologies [43]. A separate study also described higher PCSK9 mRNA and protein in the fibrotic human liver obtained from patients with hepatitis B infection and patients with non-viral disease etiology [44].

Associations of hepatic PCSK9 protein with lobular inflammation and hepatocyte ballooning were, however, not observed in patients with non-alcoholic fatty liver disease (NAFLD) [45]. Serum PCSK9 was nevertheless positively correlated with steatosis grade, necroinflammation, hepatocyte ballooning, and fibrosis stage in the second cohort of patients with NAFLD. Of note, hepatic PCSK9 expression was also associated with liver steatosis. Correlation of systemic PCSK9 and hepatic expression of lipogenic genes suggested that PCSK9 is induced in parallel with de novo lipogenesis [46]. Correlations of serum PCSK9, LDL and triglycerides did not exist in that study group. Of note, there was a highly significant inverse association of serum PCSK9 with BMI in this cohort [46].

Moreover, hepatic PCSK9 protein was not induced in liver cirrhotic patients of a further investigation, and this applied to patients with HCV or HBV infection and patients with non-viral disease etiology [10].

A sample size of studies analyzing hepatic PCSK9 protein expression was rather small. Thus, further research in larger populations must resolve the complex interplay between PCSK9 levels in serum, hepatic synthesis, liver disease severity and disease etiology.

Here, confounding variables like obesity, liver steatosis and obesity must be considered. The role of PCSK9 in glucose homeostasis and diabetes is still controversial [47]. At least in our cohort of HCV patients, serum PCSK9 was not induced in patients with diabetes. PCSK9 did not correlate with BMI in the HCV patients. Separate studies reported negative and positive associations between PCSK9 and BMI [46,48]. Serum PCSK9 was not induced in HCV patients with liver steatosis but was higher in patients with metabolic fatty liver [46]. These contradictory results show that the association of PCSK9 with metabolic traits is far from being resolved.

Inflammation in HCV infection has a critical role in liver disease progression [49]. Correlations of PCSK9 with CRP and leukocyte count were described before [47]. PCSK9 expression was enhanced in inflammation, and subsequent degradation of the hepatic LDL-receptor hindered the clearance of bacterial lipids, such as lipopolysaccharide [47]. In our study, PCSK9 was positively correlated with leukocyte count in cirrhotic patients. Interestingly, a negative association of CRP and PCSK9 existed in HCV-infected patients with cirrhosis before therapy and at 12 weeks posttreatment. CRP is produced by the liver as an acute-phase protein, and impaired IL-6 signaling, lower hepatocyte synthesis and increased inflammation may regulate CRP levels in HCV infection [50]. Inhibitory monoclonal PCSK9 antibodies did not reduce highly sensitive CRP levels in patients with an increased risk for cardiovascular diseases [51]. Infusion of endotoxin-induced CRP, but not PCSK9 in healthy volunteers [52]. These findings demonstrate that PCSK9 levels are not directly related to CRP [51].

Serum PCSK9 positively correlated with viral load in the subgroup of non-cirrhotic patients confirming a previous study where a positive correlation of HCV viral titer and PCSK9 levels was described [22]. This suggests that HCV may induce serum PCSK9 levels, and HCV infection indeed enhanced hepatic PCSK9 protein [10]. Accordingly, highly efficient eradication of HCV by DAA therapy was linked with a reduction of serum PCSK9 levels already four weeks after the start of treatment. This is in line with a further analysis, where total PCSK9 levels declined four weeks after the start of DAA treatment [27]. In contradiction to these findings, a separate study reported on a rise of PCSK9 in HCV patients with a sustained virologic response [53]. Patients were treated with interferon-based therapies, which were combined with DAAs for a not further specified number of patients [53]. Thus, the effect of HCV eradication on systemic PCSK9 levels is still an unresolved issue.

A main function of PCSK9 is to regulate LDL, and high PCSK9 is associated with high LDL [54]. Indeed, positive associations of PCSK9, cholesterol, LDL and triglycerides were identified in large cohorts [37]. Triglycerides were not documented in our patient cohort, and this is a limitation of our study. Serum PCSK9 did not correlate with LDL levels in cirrhotic and non-cirrhotic HCV-infected patients. Similarly, there were no associations of plasma PCSK9 and LDL in genotype 3- and 1-infected HCV patients [24]. Levels of active PCSK9 did not correlate with LDL in HCV patients [27]. Liver function is the main regulator of serum cholesterol levels, and impaired hepatic lipoprotein release seems to be causal for low LDL levels in patients with severe liver diseases [23]. There was, however, a modest positive correlation between PCSK9 and LDL in cirrhotic HCV patients at 12 weeks posttreatment. Such an association was not observed in the non-cirrhotic patients. Whether the functional role of PCSK9 in LDL metabolism was restored in severely ill patients after HCV elimination needs further research.

High LDL is a risk factor for cardiovascular diseases, and statins are commonly used drugs to treat hypercholesterinemia [55,56]. Statins are well tolerated in patients with compensated liver cirrhosis. In statin-intolerant patients, PCSK9 inhibitors may be used to lower LDL [57]. There are still concerns about the adverse effects of statins in patients with decompensated liver cirrhosis [55,56], and inhibition of PCSK9 may be an alternative approach. Drug–drug interactions of statins and DAAs have been described [56] and PCSK9 inhibitors may be better suited during DAA therapy [57]. Considering the anti-inflammatory and antifibrotic effects of PCSK9 inhibition, blockage of PCSK9 may have multiple beneficial effects in patients with liver cirrhosis [44,57]. Statins also decrease liver cirrhosis incidence [58], and further studies must evaluate whether inhibition of PCSK9 is of advantage. There is some evidence for the role of PCSK9 in HCV infection [18,20], and PCSK9 inhibitors may have antiviral effects. Whether serum PCSK9 levels are associated with chronic HCV infection risk must be evaluated in the future. The rapid decline of PCSK9 after viral elimination suggests a direct effect of the virus on PCSK9 serum levels. The underlying pathways and the role of liver cirrhosis on the crosstalk between HCV and PCSK9 levels must be evaluated in detail.

The study’s limitation was the small sample size, and the association of viral genotype and serum PCSK9 needs further studies. Regarding lipids, this study focused on LDL levels, and correlations with further lipoproteins, fatty acids or triglycerides were not evaluated. Moreover, the ELISA used did not discriminate between active and inactive PCSK9. Study strength was the comparison of HCV-infected and non-infected cohorts. Moreover, serum PCSK9 was measured at different time points during DAA therapy.

## 5. Conclusions

Positive associations of serum PCSK9 with viral load and decline after viral eradication in non-cirrhotic HCV patients indicate a role of HCV infection to regulate serum PCSK9. In HCV patients with liver cirrhosis, PCSK9 was low and was associated with markers of liver injury. PCSK9 levels did, accordingly, not decline in patients with liver cirrhosis after DAA therapy, which cannot improve liver function within a short time of observation. PCSK9 and LDL were changed in the opposite direction after DAA therapy. Thus, PCSK9 did not contribute to the recovery of LDL levels after the elimination of HCV. Liver function and HCV infection seem to affect serum PCSK9 and LDL levels. Thus, LDL and PCSK9 normalize after viral elimination in patients with “good” liver function. In cirrhotic patients, the impact of impaired liver function may override the effects of viral eradication.

## Figures and Tables

**Table 1:** Laboratory parameters of the HCV-infected patients and the non-infected control cohort, which included patients without severe liver diseases.

	HCV	Non-HCV	*p*-Value
Number	82	55	ns
Sex (female/male)	33/49	23/32	ns
Age (years)	59 (24–80)	58 (21–80)	ns
BMI (kg/m^2^)	27.1 (18.4–40.4)	26.3 (17.8–39.7)	ns
MELD score	8 (6–20)	na	
ALT (U/L)	86 (22–287)	32 (16–288) ^54^	<0.001
AST (U/L)	75 (17–1230)	24 (12–256) ^54^	<0.001
Albumin (g/L)	36 (19–45) ^81^	47 (32–56)	<0.001
Bilirubin (mg/dL)	0.8 (0.3–4.3)	0.5 (0.2–2.8) ^53^	<0.001
INR	1.13 (0.91–2.44)	nd	
Creatinine (mg/dL) Ferritin (ng/mL)	0.78 (0.14–1.31) ^81^ 141 (7.0–2309) ^76^	nd nd	
Leukocytes (n/L)	5.9 (2.2–72.4)	nd	
CRP (mg/L)	2.9 (2.9–72.4)	nd	
LDL (mg/dL)	88 (31–210) ^77^	112 (44–340)	<0.01
HDL (mg/dL)	51 (19–103) ^77^	nd	
Viral load (U/mL)	1.4 × 10^6^	na	
Genotype 1a/1b/3a/rare	24/38/14/6	na	
FIB-4 (no fibrosis/intermediate values/yes)	14/15/53	na	
APRI (no fibrosis/no reliable values/fibrosis/cirrhosis)	13/12/29/28	na	
ARFI (F1/F3/F4)	15/25/42	na	
NAFLD (no fibrosis/no reliable values/fibrosis/nd)	17/22/35/8	na	
PCSK9 (ng/mL)	218 (99–418)	170 (40–360)	<0.01

ALT: alanine aminotransferase; ARFI: acoustic radiation force impulse; AST: aspartate aminotransferase; BMI: body mass index; CRP: C-reactive protein; HCV: hepatitis C virus; HDL: high-density lipoprotein; INR: international normalized ratio; LDL: low-density lipoprotein; NAFLD: non-alcoholic fatty liver disease; na: not applicable; nd: not defined; ns: not significant; PCSK9: proprotein convertase subtilisin/kexin 9; In case that the laboratory values were not documented for the whole cohort, the respective numbers are given in superscript.

**Table 2:** Laboratory parameters of the cirrhotic patients with HCV and non-viral disease etiology. *** *p* < 0.001.

	HCV Cirrhosis	Non-HCV Cirrhosis
Number	37	26
Sex (female/male)	15/22	5/21
Age (years)	61 (38–80)	49 (40–81)
MELD score	10 (7–20)	8 (6–21) ^22^
Albumin (g/L)	34 (19–45)	32 (2–4)
Bilirubin (mg/dL)	1.2 (0.4–4.3)	1.1 (0.4–3.7)
LDL (mg/dL) PCSK9 (ng/mL)	68 (31–152) 171 (99–418)	35 (17–81) 108 (41–290) ***

In case that the laboratory values were not documented for the whole cohort, the respective number is given in superscript.

**Table 3:** Spearman correlation coefficients and *p*-values for the correlations of PCSK9 with body mass index (BMI), age, model for end-stage liver disease (MELD) score, viral load and routine laboratory parameters in the HCV patients before direct-acting antiviral (DAA) therapy, at 4 and 12 weeks after the start of therapy and 12 weeks posttreatment.

Parameter	Baseline (82 Patients)	4-Weeks (79 Patients)	12-Weeks (81 Patients)	12 Weeks Posttreatment (76 Patients)
BMI, kg/m^2^	0.042 (0.720)	0.037 (0.756)	0.001 (0.995)	−0.026 (0.840)
Age	**−0.248 (0.024)**	**−0.336 (0.002)**	−0.180 (0.108)	−0.153 (0.221)
MELD score	**−0.623 (<0.001)**	**−0.579 (<0.001)**	**−0.573 (<0.001)**	**−0.570 (<0.001)**
ALT, U/L	−0.021 (0.853)	−0.042 (0.711)	−0.082 (0.467)	−0.060 (0.627)
AST, U/L	**−0.260 (0.018)**	**−0.258 (0.022)**	**−0.288 (0.009)**	**−0.339 (<0.004)**
Bilirubin, mg/dL	**−0.506 (<0.001)**	**−0.587 (<0.001)**	**−0.597 (<0.001)**	**−0.568 (<0.001)**
Albumin, g/L	**0.397 (<0.001)**	**0.460 (<0.001)**	**0.527 (<0.001)**	**0.441 (<0.001)**
INR	**−0.595 (<0.001)**	**−0.549 (<0.001)**	**−0.608 (<0.001)**	**−0.574 (<0.001)**
Creatinine, mg/dL	0.074 (0.512)	−0.013 (0.909)	0.010 (0.932)	0.159 (0.191)
Leukocytes, n/L	**0.389 (<0.001)**	**0.358 (0.001)**	**0.503 (<0.001)**	**0.383 (0.001)**
CRP, mg/L HDL, mg/dL LDL, mg/dL	**−0.288 (0.009)** 0.047 (0.686) **0.502 (<0.001)**	0.218 (0.052) 0.042 (0.720) **0.513 (<0.001)**	**−0.235 (0.036)** −0.048 (0.674) **0.578 (<0.001)**	0.018 (0.885) 0.022 (0.862) **0.595 (<0.001)**
Viral load	**0.298 (0.007)**	na	na	na

Significant correlations are in bold. Laboratory parameters were documented for at least 95% of the patients; na: not applicable.

**Table 4:** Spearman correlation coefficients and *p*-values (in brackets) for the correlations of PCSK9 with age, MELD score, viral load and routine laboratory parameters in the HCV patients with and without liver cirrhosis before direct-acting antiviral (DAA) therapy, and 12 weeks posttreatment.

Parameter	Baseline (No Cirrhosis; 45 Patients)	Baseline (Cirrhosis; 37 Patients)	12 Weeks Posttreatment (No Cirrhosis; 45 Patients)	12 Weeks Posttreatment (Cirrhosis; 37 Patients)
Age	−0.010 (0.949)	−0.300 (0.071)	−0.168 (0.306)	−0.021 (0.913)
MELD score	−0.061 (0.689)	**−0.707 (<0.001)**	−0.195 (0.234)	**−0.687 (<0.001)**
ALT, U/L	−0.019 (0.900)	**−0.332 (0.045)**	−0.034 (0.836)	−0.009 (0.964)
AST, U/L	0.063 (0.681)	−0.011 (0.951)	0.047 (0.776)	−0.323 (0.082)
Bilirubin, mg/dL	−0.097 (0.525)	**−0.653 (<0.001)**	−0.294 (0.069)	**−0.603 (0.001)**
Albumin, g/L	−0.117 (0.451)	**0.442 (0.006)**	**−0.322 (0.049)**	0.283 (0.130)
INR	−0.083 (0.589)	**−0.567 (<0.001)**	−0.180 (0.274)	**−0.567 (<0.001)**
Leukocytes, n/L	−0.052 (0.735)	**0.495 (0.002)**	−0.083 (0.615)	0.367 (0.050)
CRP, mg/L LDL, mg/dL	−0.185 (0.223) 0.207 (0.193)	**−0.526 (0.001)** 0.316 (0.061)	−0.218 (0.182) 0.270 (0.123)	**−0.526 (0.001)** **0.459 (0.014)**
Viral load	**0.326 (0.029)**	−0.003 (0.987)	na	na

Laboratory parameters were documented for at least 95% of the patients. Significant correlations are in bold (not applicable, na).

## Data Availability

The data presented in this study are available on request from the corresponding author.

## Supplementary Materials

The following are available online at https://www.mdpi.com/article/10.3390/jcm10081621/s1.
