# Supplementary material for: Rapid Decline of Serum Proprotein Convertase Subtilisin/Kexin 9 (PCSK9) in Non-Cirrhotic Patients with Chronic Hepatitis C Infection Receiving Direct-Acting Antiviral Therapy
Source: J Clin Med. 2021 Apr 11;10(8):1621. doi: 10.3390/jcm10081621 (PMC8069657; doi:10.3390/jcm10081621)
Supplement: Supplementary file 1 [file jcm-10-01621-s001.pdf]

Experiment 1

Using Standard Data Set from Current Experiment.

4-Parameters Fit: Y=(A-D)/(1+(X/C)^B)+D

20/50/80%: X = 1617,957 / 5582,148 / 19259,089   Y = 0,509 / 1,265 / 2,020

A: 0,005 (+/-0,003), B: 1,119 (+/-0,023), C: 5582,148 (+/-334,220), D: 2,524 (+/-0,079)

chi2=0,001, RMS=0,008, r^2=1,000

Standards Report:

Std #	Conc	Well	Replicate	Mean	SD
1	38	G1	0,009	0,003	0,008
		G2	-0,003		
2	82	F1	0,033	0,029	0,006
		F2	0,025		
3	204.8	E1	0,089	0,079	0,014
		E2	0,069		
4	512.0	D1	0,188	0,167	0,03
		D2	0,146		
5	1280.0	C1	0,43	0,404	0,036
		C2	0,379		
6	3200.0	B1	0,9	0,889	0,016
		B2	0,877		
7	8000	A1	1,579	1,514	0,092
		A2	1,449		

Sample Report:

Sample ID	Well	Replicates	Mean	Conc	SD (Conc)	Conc pg/ml	Serum Nr	%CV
S01	A3	1,03	1,015	3899,239	122,153	38992,39	1 (1:10)	3,13
	A4	1						
S02	B3	1,097	1,106	4449,124	77,106	44491,24	2 (1:10)	1,73
	B4	1,114						
S03	C3	1,32	1,318	6026,37	18,17	60263,7	3 (1:10)	0,30
	C4	1,317						
S04	D3	0,989	1,001	3822,105	100,233	38221,05	4 (1:10)	2,62
	D4	1,014						
S05	E3	0,733	0,728	2475,724	30,344	24757,24	5 (1:10)	1,23
	E4	0,723						
S06	F3	0,767	0,748	2560,151	120,481	25601,51	6 (1:10)	4,71
	F4	0,728						
S07	G3	1,095	1,088	4334,939	66,546	43349,39	7 (1:10)	1,54
	G4	1,08						
S08	H3	1,066	1,031	3995,154	286,474	39951,54	8 (1:10)	7,17
	H4	0,997						
S09	A5	0,217	0,213	647,532	19,35	32376,6	1 (1:50)	2,99
	A6	0,208						
S10	B5	0,399	0,418	1301,977	90,554	65098,85	2 (1:50)	6,96
	B6	0,437						
S11	C5	0,454	0,46	1443,739	26,941	72186,95	3(1:50)	1,87

	C6	0,465						
S12	D5	0,243	0,249	759,197	26,133	37959,85	4 (1:50)	3,44
	D6	0,255						
S13	E5	0,177	0,181	552,166	17,062	27608,3	5 (1:50)	3,09
	E6	0,185						
S14	F5	0,172	0,18	550,658	36,255	27532,9	6 (1:50)	6,58
	F6	0,189						
S15	G5	0,228	0,243	740,739	65,187	37036,95	7 (1:50)	8,80
	G6	0,258						
S16	H5	0,302	0,312	957,5	47,076	47875	8 (1:50)	4,92
	H6	0,323						
S17	A7	0,049	0,053	164,53	17,729	32906	1 (1:200)	10,78
	A8	0,057						
S18	B7	0,157	0,163	497,961	25,518	99592,2	2 (1:200)	5,12
	B8	0,169						
S19	C7	0,144	0,145	442,377	2,123	88475,4	3 (1:200)	0,48
	C8	0,145						
S20	D7	0,07	0,07	217,245	0	43449	4 (1:200)	0,00
	D8	0,07						
S21	E7	0,058	0,06	184,808	6,59	36961,6	5 (1:200)	3,57
	E8	0,061						
S22	F7	0,052	0,056	173,911	17,654	34782,2	6 (1:200)	10,15
	F8	0,06						
S23	G7	0,064	0,066	203,386	6,547	40677,2	7 (1:200)	3,22
	G8	0,067						
S24	H7	0,099	0,097	299,469	8,553	59893,8	8 (1:200)	2,86
	H8	0,095						
						High	%CV	2,80
						Median	%CV	4,83
						Low	%CV	4,52

Experiment 2

Using Standard Data Set from Current Experiment.

4-Parameters Fit: Y=(A-D)/(1+(X/C)^B)+D

20/50/80%: X = 2306,356 / 9858,426 / 42139,437   Y = 0,626 / 1,562 / 2,498

A: 0,001 (+/-0,001), B: 0,954 (+/-0,009), C: 9858,426 (+/-461,676), D: 3,122 (+/-0,075)

chi2=0,000, RMS=0,003, r^2=1,000

Standards Report:

Std #	Conc	Well	Replicate	Mean	SD
1	38	G1	0,012	0,01	0,002
		G2	0,009		
2	82	F1	0,04	0,036	0,006
		F2	0,032		
3	204.8	E1	0,087	0,081	0,008
		E2	0,076		
4	512.0	D1	0,188	0,174	0,021
		D2	0,159		
5	1280.0	C1	0,407	0,39	0,024
		C2	0,373		
6	3200.0	B1	0,894	0,797	0,137
		B2	0,7		
7	8000	A1	1,445	1,406	0,054
		A2	1,368		

Sample Report:

Sample ID	Well	Replicates	Mean	Conc	SD (Conc)	Conc pg/ml	Serum Nr	%CV
S01	A3	0,902	0,864	3593,632	328,704	35936,32	1 (1:10)	9,15
	A4	0,825						
S02	B3	1,024	0,995	4440,553	281,836	44405,53	2 (1:10)	6,35
	B4	0,966						
S03	C3	1,236	1,202	6031,971	405,447	60319,71	3 (1:10)	6,72
	C4	1,169						
S04	D3	0,884	0,86	3575,552	200,02	35755,52	4 (1:10)	5,59
	D4	0,837						
S05	E3	0,678	0,656	2453,528	158,247	24535,28	5 (1:10)	6,45
	E4	0,633						
S06	F3	0,689	0,654	2448,557	242,455	24485,57	6 (1:10)	9,90
	F4	0,62						
S07	G3	0,998	0,989	4399,454	86,922	43994,54	7 (1:10)	1,98
	G4	0,98						
S08	H3	1,033	1,014	4568,859	193,081	45688,59	8 (1:10)	4,23
	H4	0,994						
S09	A5	0,194	0,207	612,509	61,434	30625,45	1 (1:50)	10,03
	A6	0,22						
S10	B5	0,343	0,35	1120,803	34,906	56040,15	2 (1:50)	3,11
	B6	0,356						
S11	C5	0,392	0,388	1269,488	22,216	63474,4	3(1:50)	1,75

	C6	0,384					
S12	D5	0,213	0,219	651,144	26,271	32557,2	4 (1:50)	4,03
	D6	0,224					
S13	E5	0,155	0,16	458,915	22,585	22945,75	5 (1:50)	4,92
	E6	0,165					
S14	F5	0,159	0,164	470,113	20,397	23505,65	6 (1:50)	4,34
	F6	0,168					
S15	G5	0,196	0,207	610,839	49,597	30541,95	7 (1:50)	8,12
	G6	0,217					
S16	H5	0,28	0,285	880,904	22,819	44045,2	8 (1:50)	2,59
	H6	0,289					
S17	A7	0,074	0,065	170,459	36,472	34091,8	1 (1:200)	21,40
	A8	0,056					
S18	B7	0,157	0,156	446,165	4,499	89233	2 (1:200)	1,01
	B8	0,155					
S19	C7	0,131	0,136	381,621	19,826	76324,2	3 (1:200)	5,20
	C8	0,14					
S20	D7	0,08	0,077	203,68	14,409	40736	4 (1:200)	7,07
	D8	0,073					
S21	E7	0,063	0,06	157,604	10,066	31520,8	5 (1:200)	6,39
	E8	0,058					
S22	F7	0,058	0,053	136,37	19,902	27274	6 (1:200)	14,59
	F8	0,048					
S23	G7	0,081	0,071	187,726	40,864	37545,2	7 (1:200)	21,77
	G8	0,061					
S24	H7	0,1	0,094	253,707	27,334	50741,4	8 (1:200)	10,77
	H8	0,087					

High	%CV	6,30
Median	%CV	4,86
Low	%CV	11,02

Experiment 3

Using Standard Data Set from Current Experiment.

4-Parameters Fit: Y=(A-D)/(1+(X/C)^B)+D

20/50/80%: X = 1831,176 / 6784,052 / 25133,226   Y = 0,513 / 1,283 / 2,053

A: -0,001 (+/-0,002), B: 1,059 (+/-0,019), C: 6784,052 (+/-446,204), D: 2,566 (+/-0,088)

chi2=0,000, RMS=0,007, r^2=1,000

Standards Report:

Std #	Conc	Well	Replicate	Mean	SD
1	38	G1	0,01	0	0,013
		G2	-0,009		
2	82	F1	0,034	0,024	0,013
		F2	0,015		
3	204.8	E1	0,078	0,072	0,008
		E2	0,066		
4	512.0	D1	0,164	0,154	0,013
		D2	0,145		
5	1280.0	C1	0,382	0,369	0,018
		C2	0,356		
6	3200.0	B1	0,878	0,801	0,109
		B2	0,724		
7	8000	A1	1,411	1,394	0,024
		A2	1,377		

Sample Report:

Sample ID	Well	Replicates	Mean	Conc	SD (Conc)	Conc pg/ml	Serum Nr	%CV
S01	A3	0,83	0,845	3465,929	122,499	34659,29	1 (1:10)	3,53
	A4	0,86						
S02	B3	1,066	1,031	4662,704	348,41	46627,04	2 (1:10)	7,47
	B4	0,997						
S03	C3	1,219	1,197	5981,169	268,961	59811,69	3 (1:10)	4,50
	C4	1,176						
S04	D3	0,848	0,838	3428,526	77,046	34285,26	4 (1:10)	2,25
	D4	0,829						
S05	E3	0,626	0,62	2306,37	35,996	23063,7	5 (1:10)	1,56
	E4	0,615						
S06	F3	0,641	0,63	2350,526	72,622	23505,26	6 (1:10)	3,09
	F4	0,619						
S07	G3	0,971	0,955	4140,823	147,571	41408,23	7 (1:10)	3,56
	G4	0,939						
S08	H3	1,005	0,989	4370,36	148,812	43703,6	8 (1:10)	3,41
	H4	0,974						
S09	A5	0,196	0,201	662,436	23,825	33121,8	1 (1:50)	3,60
	A6	0,206						
S10	B5	0,357	0,356	1210,521	5,267	60526,05	2 (1:50)	0,44
	B6	0,355						
S11	C5	0,347	0,35	1186,368	13,107	59318,4	3(1:50)	1,10

	C6	0,352						
S12	D5	0,185	0,19	625,485	23,684	31274,25	4 (1:50)	3,79
	D6	0,195						
S13	E5	0,133	0,14	458,428	30,064	22921,4	5 (1:50)	6,56
	E6	0,146						
S14	F5	0,144	0,149	489,558	23,218	24477,9	6 (1:50)	4,74
	F6	0,154						
S15	G5	0,184	0,187	615,444	14,188	30772,2	7 (1:50)	2,31
	G6	0,19						
S16	H5	0,262	0,268	894,389	32,246	44719,45	8 (1:50)	3,61
	H6	0,275						
S17	A7	0,048	0,044	151,446	15,996	30289,2	1 (1:200)	10,56
	A8	0,041						
S18	B7	0,155	0,151	496,128	18,591	99225,6	2 (1:200)	3,75
	B8	0,147						
S19	C7	0,116	0,115	380,294	2,292	76058,8	3 (1:200)	0,60
	C8	0,115						
S20	D7	0,068	0,065	217,489	13,644	43497,8	4 (1:200)	6,27
	D8	0,062						
S21	E7	0,052	0,05	169,2	9,121	33840	5 (1:200)	5,39
	E8	0,048						
S22	F7	0,051	0,048	162,748	13,691	32549,6	6 (1:200)	8,41
	F8	0,045						
S23	G7	0,067	0,061	204,623	27,3	40924,6	7 (1:200)	13,34
	G8	0,055						
S24	H7	0,095	0,088	293,082	29,605	58616,4	8 (1:200)	10,10
	H8	0,082						
						High	%CV	3,67
						Median	%CV	3,27
						Low	%CV	7,30

Intra-assay		
High	4,25666008	%CV
Median	4,31981966	%CV
Low	7,61668716	%CV

Inter-Assay		
High	3,40532807	%CV
Medium	3,45585573	%CV
Low	6,09334973	%CV
